# Modulated Electro-Hyperthermia as Palliative Treatment for Pancreatic Cancer: A Retrospective Observational Study on 106 Patients

**DOI:** 10.1177/1534735419878505

**Published:** 2019-09-27

**Authors:** Giammaria Fiorentini, Donatella Sarti, Virginia Casadei, Carlo Milandri, Patrizia Dentico, Andrea Mambrini, Roberto Nani, Caterina Fiorentini, Stefano Guadagni

**Affiliations:** 1 Azienda Ospedaliera “Ospedali Riuniti Marche Nord,” Pesaro, Italy; 2 Nuovo Ospedale San Giuseppe, Empoli, Florence, Italy; 3 Apuane General Hospital, Carrara, Italy; 4 University of Milano Bicocca, ASST Papa Giovanni XXIII, Bergamo, Italy; 5 University of Siena, Siena, Italy; 6 University of L’Aquila, L’Aquila, Italy

**Keywords:** pancreatic cancer, modulated electro-hyperthermia, survival, tumor response, gemcitabine, FOLFIRINOX

## Abstract

**Background:** Pancreatic adenocarcinoma has a poor prognosis, resulting in a <10% survival rate at 5 years. Modulated electro-hyperthermia (mEHT) has been increasingly used for pancreatic cancer palliative care and therapy. **Objective:** To monitor the efficacy and safety of mEHT for the treatment of advanced pancreatic cancer. **Methods:** We collected data retrospectively on 106 patients affected by stage III-IV pancreatic adenocarcinoma. They were divided into 2 groups: patients who did not receive mEHT (no-mEHT) and patients who were treated with mEHT. We performed mEHT applying a power of 60 to 150 W for 40 to 90 minutes. The mEHT treatment was associated with chemotherapy and/or radiotherapy for 33 (84.6%) patients, whereas 6 (15.4%) patients received mEHT alone. The patients of the no-mEHT group received chemotherapy and/or radiotherapy in 55.2% of cases. **Results:** Median age of the sample was 65.3 years (range = 31-80 years). After 3 months of therapy, the mEHT group had partial response in 22/34 patients (64.7%), stable disease in 10/34 patients (29.4%), and progressive disease in 2/34 patients (8.3%). The no-mEHT group had partial response in 3/36 patients (8.3%), stable disease in 10/36 patients (27.8%), and progressive disease in 23/36 patients (34.3%). The median overall survival of the mEHT group was 18.0 months (range = 1.5-68.0 months) and 10.9 months (range = 0.4-55.4 months) for the non-mEHT group. **Conclusions:** mEHT may improve tumor response and survival of pancreatic cancer patients.

## Introduction

Pancreatic cancer has a very poor prognosis with a median survival of 4.6 months and overall survival (OS) 3% at 5 years.^1,2^ It is the fourth leading cause of cancer death in Europe, and 85% of patients affected by pancreatic cancer are already in progression or advanced stage of disease at diagnosis.^3,4^ Pancreatic carcinoma is the 14th most common cancer worldwide and has the seventh highest mortality.^5^ Its largest incidence is in Europe and the smallest in South-Central Asia.^6^

The efficacy of neoadjuvant chemo-radiotherapy association is still not clear. Exploratory laparotomy followed by resection and adjuvant chemotherapy are the first-line therapy in cases of resectable disease, resulting in a better prognosis.^7-10^ Most patients, however, are not resectable or develop recurrence early after surgery.^2^ In these cases, gemcitabine-based chemotherapy is the most common treatment via systemic or regional intra-arterial infusion.^11^

The FOLFIRINOX schedule (leucovorin, fluorouracil, irinotecan, and oxaliplatin) is indicated for fit patients presenting locally advanced or metastatic pancreatic cancer and shows encouraging results.^12,13^ Other therapies for locally advanced disease are radiofrequency ablation, stereotactic body radiation therapy, and irreversible electroporation; however, their efficacy has not yet been confirmed by randomized studies.^14-16^ The combination of gemcitabine with cisplatin^17^ and gemcitabine with nab-paclitaxel also results in improved survival for metastatic pancreatic tumor.^2,18^

Hyperthermia can be used as cancer therapy, and it allows temperatures of 39°C to 43°C inside tumor mass. It is typically a complementary treatment, often used in association with chemotherapy and/or radiotherapy, increasing their efficacy and prolonging their clinical benefits.^19,20^ A recent review analyzed 1294 articles and selected 14 most relevant articles showing the benefits of hyperthermia.^21^ The application of immunotherapy in combination with hyperthermia also has beneficial effects.^22-24^

The benefits of hyperthermia combined with chemotherapy are due to heat-induced improvement in drug delivery, increase in blood flow and oxygen radical production,^25^ inhibition of hypoxia,^26^ angiogenesis, and DNA repair, resulting in enhanced tumor cell death.^27,28^ The combination of hyperthermia with chemotherapy or radiotherapy is successful in several types of tumors, such as esophageal, breast, brain, and pancreatic cancers.^21,29-36^

Modulated electro-hyperthermia (mEHT) is a type of hyperthermia that is more selective in killing tumor cells,^37^ while sparing healthy cells^38^ and overcoming the limited penetration of radiofrequency (13.56 MHz) in human tissues.^39^ The temperature inside the tissues cannot be measured directly but it can be estimated from input power,^40^ due to the high efficacy^41^ and the synergy of the electric field.^42^ The targeted malignant cells absorb the heat that raises their temperature >3°C than their environment.^43^ In this way, malignant cells may achieve temperatures of 39°C to 43°C.^44^

Clinical data show that mEHT is feasible not only for palliative care but also for therapeutic purposes in advanced cancer, offering the potential to prolong OS and improve quality of life.^45,46^ Several studies show advantages and curative effects of mEHT alone or in association with chemo-radiotherapy for advanced pancreas carcinoma.^47-50^

In this study, the effect of mEHT is monitored in terms of tumor response, OS, and safety in locally advanced or metastatic pancreatic adenocarcinoma.

## Materials and Methods

### Patient Selection

This is a retrospective observational multicentric study on the efficacy and safety of mEHT for advanced pancreatic cancer therapy. Patients were included in the study if they had diagnosis of advanced stage (III-IV) pancreatic adenocarcinoma, they were >18 years old, had signed the informed consent, their Eastern Cooperative Oncology Group (ECOG) performance status was ≥2, and they had normal hematological parameters. Patients were excluded from the study if they had a pacemaker, bilirubin, or transaminase levels >3 times the normal value upper range level or bleeding.

From April 2013 to March 2019, 170 patients with advanced or relapsed pancreatic cancer were screened in 3 Italian hospitals; 106 of these patients met the inclusion criteria and were enrolled in the study. Data were evaluated retrospectively from diagnosis to death or last follow-up of patients.

The sample was divided into 2 comparative groups: patients who did not receive mEHT (no-mEHT, 67/106, 63.2%) and patients who were treated with mEHT (39/106, 36.8%). mEHT was performed in association with chemotherapy in 32 (82%) of patients, whereas 7 (18%) received mEHT alone.

The majority (54%) of no-mEHT group received a second-line chemotherapy, whereas 31 (46%) received integrative and supportive care (vitamins, analgesics, parenteral nutrition, acupuncture, and phytotherapy).

### mEHT Protocol and Device

Modulated electro-hyperthermia was performed using the EHY-2000plus device (CE0123, Oncotherm, Torisdorf, Germany), applying a radiofrequency current of 13.56 MHz as carrier frequency^51^ that was modulated by time-fractal fluctuation.^52^ The energy was transferred by capacitive coupling, with precise impedance matching.^53^

The selected upper abdominal quadrant was treated for a median of 3 sessions per week, for a total of 8 weeks, increasing the power applied and length of each session. The first mEHT treatment was always performed applying 60 W for 40 minutes, then the time was gradually raised to 90 minutes and the power to 150 W in 2 weeks. The treatment was prolonged if there was evidence of positive effects.^32,43,54,55^

Patients treated with chemotherapy were treated with mEHT the same day or within the following 48 hours. During this period of time, indeed, the blood concentration of chemotherapy drugs is still high enough to benefit from mEHT synergy.

### Outcome Measures

Magnetic resonance imaging or computed tomography scan was performed every 3 months after first-line therapy and following therapy lines, including mEHT. Tumor response was assessed using RECIST (Response Evaluation Criteria in Solid Tumors) version 1.1. Functional recovery was assessed using the ECOG Performance Status scale; in particular, a reduction of 1 point in the scale was considered as positive functional improvement.

Overall survival was computed from diagnosis date to last follow-up or death of the patient in both groups. CTCAE (Common Terminology Criteria for Adverse Events) version 3.0 was used to classify type and intensity of adverse events.

### Statistical Analysis

Continuous data were reported as median and ranges and proportions as percentages. OS was graphically represented using Kaplan-Meier nonparametric estimates with survival probability on the vertical axis and time from diagnosis (in months) on the horizontal axis. Student’s *t* test, *z* test for proportions, and log-rank test for Kaplan-Meier curves were used for the assessment of statistical significance with *P* ≤ .05 taken to indicate statistically significant differences.

## Results

### The Sample

The sample included 106 consecutive patients with a median age of 65.3 years (range = 31-80 years; Table 1). The gender distribution was 59 (55.7%) males and 47 (44.3%) females. Many patients (58.5%) developed metastases. The most frequent metastatic site was the liver (75.8%), and 6.5% of the patients had multiple hepatic lesions (Table 1). First-line chemotherapy was administered to 99 (93.4%) patients, surgery to 22 (20.8%) patients, and radiotherapy to 8 (7.5%) patients. The first-line chemotherapy was mainly based on gemcitabine alone or in combination with other drugs (Table 2).

**Table 1.** Description of the Sample.

Ages	All Patients		With mEHT		Without mEHT	
	Mean	Median	Mean	Median	Mean	Median
Average age (years)	64.5	65.3	61.8	62.6	66	67.8
Groups	All Patients		With mEHT		Without mEHT	
	n	%	n	%	n	%
Males	59	55.7	24	61.5	38	56.7
Females	47	44.3	15	38.5	29	43.3
Non-metastatic	44	41.5	14	35.9	30	44.8
Metastatic	62	58.5	25	64.1	37	55.2
Site of Metastases	All Patients		With mEHT		Without mEHT	
	n	%	n	%	n	%
Liver	47	75.8	19	76.0	28	75.7
Multiple site	4	6.5	4	16.0	0	0.0
Lung	5	8.1	1	4.0	4	10.8
Lymph nodes	2	3.2		0.0	2	5.4
Peritoneum	1	1.6		0.0	1	2.7
Bones	2	3.2		0.0	2	5.4
Pelvis	1	1.6	1	4.0		0.0

Abbreviation: mEHT, modulated electro-hyperthermia.

**Table 2.** Types of First-Line Chemotherapy.

Type of First-Line Chemotherapy	All Patients		With mEHT		Without mEHT	
	n	%	n	%	n	%
Gemcitabine oxaliplatin	49	46.2	14	35.9	35	52.2
Gemcitabine	30	28.3	6	15.4	24	35.8
Gemcitabine Abraxane	8	7.5	5	12.8	3	4.5
Gemcitabine FU	4	3.8	2	5.1	2	3.0
Other	8	7.5	5	12.8	3	4.5
No	7	6.6	7	17.9	0	0.0

Abbreviations: mEHT, modulated electro-hyperthermia, FU, 5-fluorouracil.

The mEHT treatment was associated with chemotherapy and/or radiotherapy for 33 (84.6%) patients, whereas 6 (15.4%) patients received mEHT alone. The patients of no-mEHT group received chemotherapy and/or radiotherapy in 55.2% of cases. Types of second-line therapies are listed in Table 3.

**Table 3.** Types of Second-Line Chemotherapy.

Continuation of Chemotherapy	All Patients		With mEHT		Without mEHT	
	n	%	n	%	n	%
Gemcitabine oxaliplatin	4	3.8	1	2.6	3	4.5
Gemcitabine-carboplatin	3	2.8	0	0.0	3	4.5
Gemcitabine abraxane	7	6.6	5	12.8	2	3.0
Gemcitabine	31	29.2	23	59.0	8	11.9
Folfiri or FOLFIRINOX	7	6.6	1	2.6	6	9.0
Folfox	8	7.5	0	0.0	8	11.9
Other	10	9.4	3	7.7	7	10.4
No	36	34.0	6	15.4	30	44.8

Abbreviation: mEHT, modulated electro-hyperthermia.

### Tumor Response

**Figure 1.**
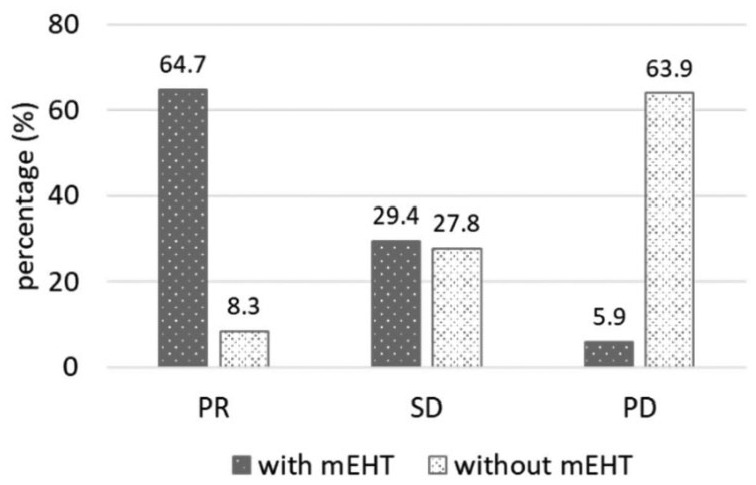
Tumor response at 3 months.PR, partial response; SD, stable disease; PD, progressive disease; mEHT, modulated electro-hyperthermia.

The analysis of tumor response was performed 3 months after mEHT + chemotherapy (mEHT group) or chemotherapy alone (no-mEHT group). Data were available for 34 and 36 patients in the mEHT and non-mEHT groups, respectively. The mEHT group had 22/34 (64.7%) partial response (PR), 10/34 (29.4%) stable disease (SD), and 2/34 (5.9%) progressive disease. As concerning the no-mEHT group, PR was observed in 3/36 (8.3%) patients, SD in 10/36 (27.8%) patients, and progressive disease in 23/36 (63.9%) patients (Figure 1).

### Survival

**Figure 2.**
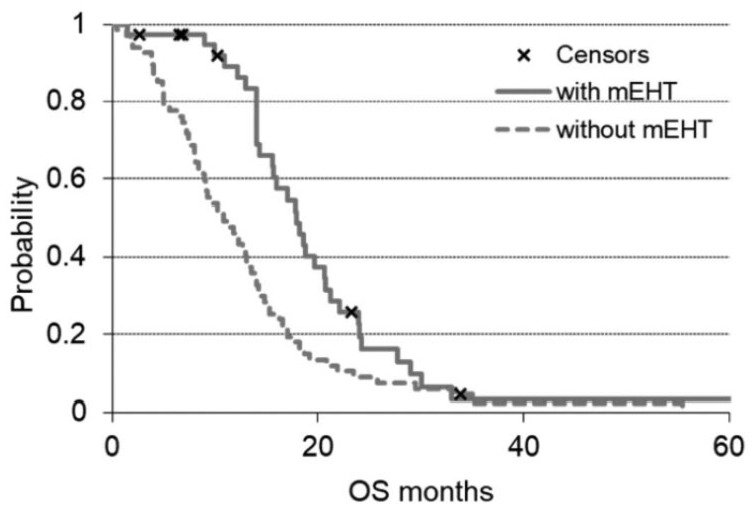
OS (overall survival) of the 2 groups of the study. The solid line is the survival of modulated electro-hyperthermia (mEHT) group and the dashed line the non-mEHT. The “x” indicates the censored patients.

The median OS of the mEHT group was 18.0 months (range = 1.5-68 months) and was significantly (*P* < .00165) higher than the 10.9 months observed (range = 0.4-55.4 months) for the non-mEHT group (Figure 2).

**Figure 3.**
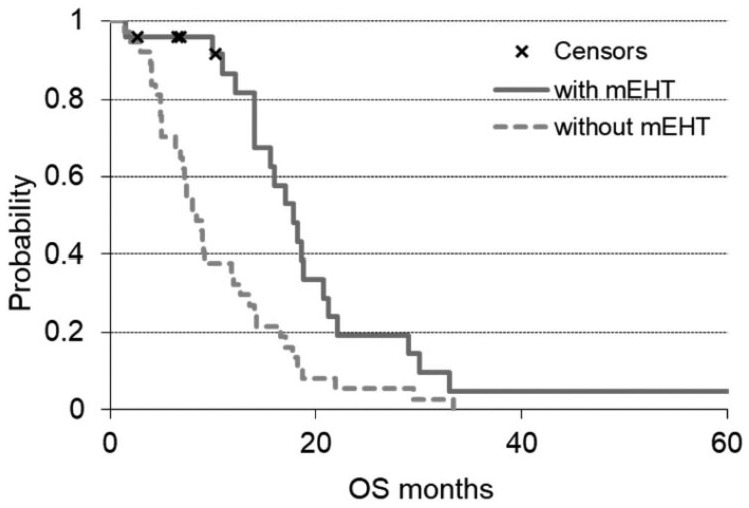
OS (overall survival) grouped by metastatic patients of the 2 groups of the study. The solid line is the survival of modulated electro-hyperthermia (mEHT) group and the dashed line the non-mEHT. The “x” indicates the censored patients.

The OS analysis of metastatic patients showed a significantly higher OS in the mEHT group (*P* = .0008). The arm with mEHT had n = 25 metastatic patients with a median OS of 17.8 months, while the non-mEHT group had n = 37 metastatic patients with median OS of 8.4 months (Figure 3).

**Figure 4.**
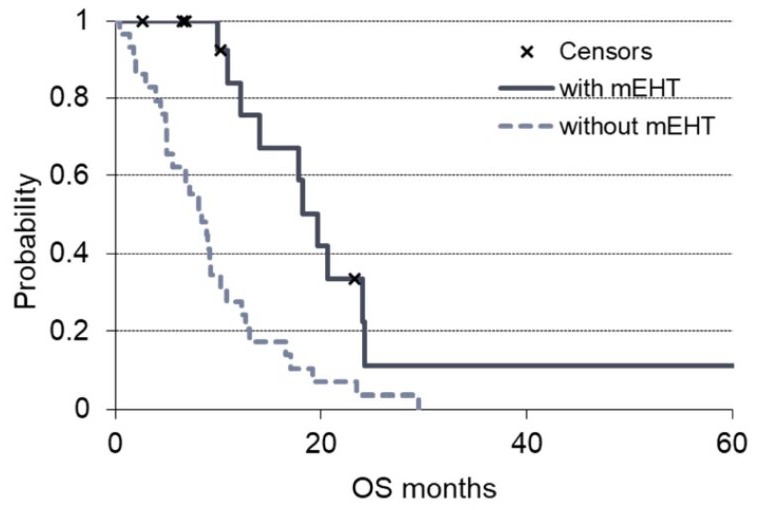
OS (overall survival) grouped by the first-line treatments of the 2 groups of the study. The solid line is the survival of modulated electro-hyperthermia (mEHT) group and the dashed line the non-mEHT. The “x” indicates the censored patients.

The benefit of mEHT in terms of survival was observed also when mEHT was used as first-line therapy (no previous treatments before mEHT). The arm with mEHT had n = 16 patients who received mEHT as first-line therapy that showed a median OS of 19.6 months, significantly (*P* = .00047) higher than that of the patients of the non-mEHT group who received only first-line chemotherapy (n = 29) and had a median OS of 8.4 months (Figure 4).

**Figure 5.**
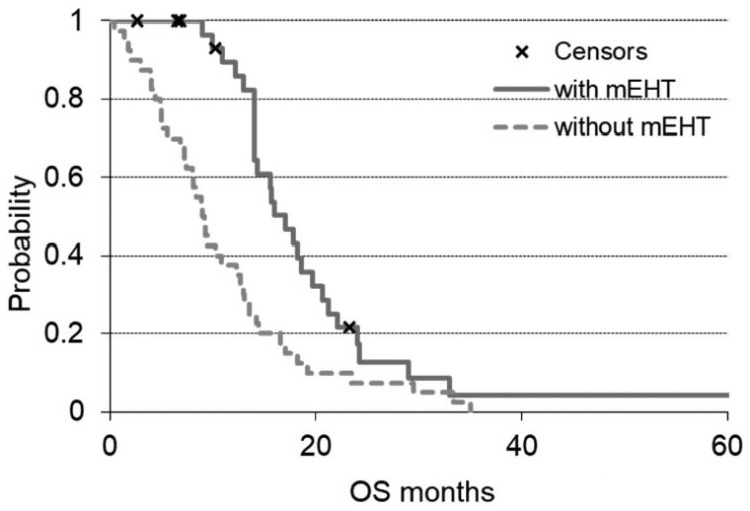
OS (overall survival) grouped for non-resected patients of the 2 groups of the study. The solid line is the survival of modulated electro-hyperthermia (mEHT) treated, the dashed line the non-mEHT treated survival curve, and the “x” indicates the censored patients.

Patients who did not undergo pancreatic surgery before mEHT therapy had a significantly higher OS than those who were not operated in the no-mEHT group (Figure 5). The arm with mEHT had n = 32 non-resected patients with a median OS of 17.0 months, while the non-mEHT group had n = 40 non-resected patients with a median OS of 9 months (*P* = .00094).

The dependent *t* test showed correlation between the time to the first mEHT treatment from the first diagnosis and the survival time from the first mEHT treatment (*P* = .46).

### Adverse Effects and Safety

Each patient received an average 12.8 (range = 2-23) sessions of mEHT. Out of a total of 499 mEHT delivered sessions, the safety assessment of mEHT showed a limited number of adverse events 20/499 (4%). mEHT toxicity consisted of skin pain in 12 (2%) sessions, grade 1 burns in 6 (1%) patients, and grade 2 burns in 2 patients. All these side effects were G1-G2 intensity and resolved with local medications and discontinuation of treatment for 1 week. All patients were evaluated before mEHT with electrocardiogram and cardiac ultrasound. No one had cardiac toxicity, increased blood pressure, or rhythm changes during mEHT treatments.

## Discussion

Efficacy of standard treatments is poor for stage III-IV pancreatic adenocarcinoma. Available therapies include surgery after neoadjuvant chemotherapy, chemotherapy with FOLFIRINOX or gemcitabine-based therapy, nab-paclitaxel, or radiotherapy.^13-16,18^ The systemic therapies, however, have a limited efficacy because of patients’ poor conditions and their severe toxicity. Hyperthermia enhances the effects of chemo-radiotherapies in pancreatic cancers, increasing overall and progression-free survival.^33-35,56,57^ mEHT allows use of a lower power than conventional hyperthermia^41,58^ and can be applied with good results for pancreatic cancer treatment.^47-50^

The tumor response analysis showed a response rate (RR = PR + SD) of 94.1% for the mEHT group and 36.1% for the non-mEHT group. A recent review on hyperthermia efficacy in pancreatic cancer therapy reported the results of 14 studies including a total of 395 patients. This article reported an overall RR of 43.9% for hyperthermia and 35.3% for the control group.^21^ The present study showed a 64.7% of PR in mEHT group that was close to the 57% and 60% reported by Kouloulias and colleagues in 2 different studies.^56,57^

The median OS of the mEHT group was 18 months (range = 1.5-68 months) and was in agreement with 18.5 months of Kouloulias et al^56^ and 18.6 months of Ohguri et al.^33^ The OS analysis of 201 patients in the 14 published studies on hyperthermia for pancreatic cancer treatment showed an overall median survival 10.5 months (range = 1-53 months),^21^ which was lower than the median OS (18 months) of mEHT group as reported in the present study. The survival curves after a certain period converge because the therapies both with and without mEHT have selected the patients with more favorable characteristics. The most important result, however, is the statistically significant difference in the first observation period (20 months), showing a potential benefit of mEHT in survival improvement of pancreatic cancer patients.

Conventional hyperthermia is mostly applied for locally advanced pancreas tumors. mEHT may be successfully applicable also for metastatic patients as suggested in the present study, where OS of metastatic patients is higher in mEHT group than in non-mEHT treated group (*P* < .00085).

The benefit of mEHT in terms of survival was also observed when mEHT was used as first-line therapy (no previous treatments before mEHT) with a median OS of 19.6 months that was significantly (*P* = .00047) higher than that of the patients of the non-mEHT group.

Non-resected patients had a significant higher OS in the mEHT group than in the no-mEHT group with a median OS of 17.0 months versus 9 months of the no-mEHT group (*P* = .00094).

A total of 499 mEHT sessions were delivered in this study, resulting in a limited number of adverse events (20/499 4%) correlated to mEHT. These adverse events (pain, burns, or discomfort) had a low intensity (G1-G2) and short duration. This low mEHT toxicity correlation has been shown also in previous studies.^21,32-35,54-56^ This may suggest a better safety of the mEHT than the conventional hyperthermia that resulted in 935 complains from 70 hyperthermia treatments as reported in a recent study.^56^

Existing hyperthermia reports are heterogeneous and methodologically different; however, they report an advantage of hyperthermia in prolonging OS and improving quality of life.^19,21,29,33-35,46^ Further randomized studies are required to confirm these findings with larger number of patients.

## Conclusion

In conclusion, longer median OS and better tumor response were observed for the mEHT group than for the control group. These results may suggest a beneficial effect of mEHT when combined with chemotherapy and/or radiotherapy, increasing response and OS for patients with locally advanced or metastatic pancreatic cancer. The results of this study suggested also that mEHT could be safe for pancreatic cancer therapy, resulting in very limited side effects.
